# Data Resource Profile: WHO Health Equity Monitor (HEM)

**DOI:** 10.1093/ije/dyw176

**Published:** 2016-09-30

**Authors:** Ahmad Reza Hosseinpoor, Nicole Bergen, Anne Schlotheuber, Cesar Victora, Ties Boerma, Aluisio JD Barros

**Affiliations:** ^1^World Health Organization, Geneva, Switzerland; ^2^University of Ottawa, Ottawa, Canada; ^3^International Center for Equity in Health, Federal University of Pelotas, Pelotas, Brazil

## Data resource basics

The Health Equity Monitor (HEM) is one component theme of the Global Health Observatory, the main statistics repository of the World Health Organization (WHO). Launched in 2013, HEM is a collaboration between: the WHO Department of Information, Evidence and Research (Geneva, Switzerland); the WHO Gender, Equity and Human Rights Team (Geneva, Switzerland); and the International Center for Equity in Health (ICEH) based in the Federal University of Pelotas (Pelotas, Brazil). HEM was created as a resource to promote and enable global and national health inequality monitoring, particularly within low- and middle-income countries, where data availability may be limiting. The practice of health inequality monitoring requires health data that are disaggregated by population subgroups (i.e. by dimensions of inequality); to this end, HEM contains high-quality, disaggregated health data that are comparable across countries and over time. Currently, reproductive, maternal, newborn and child health (RMNCH) is the featured topic of HEM, which contains indicators categorized under the following sub-themes: reproductive health interventions; maternal health interventions; newborn and child health interventions; RMNCH interventions (composite index); and health outcomes. Data are disaggregated by dimensions of inequality including education, economic status, place of residence, subnational region and child's sex (where applicable).

The two main components of HEM are the data repository and the theme page. The HEM data repository contains re-analysed (secondary) data taken from large-scale, nationally representative household health surveys: Demographic and Health Surveys (DHS) and Multiple Indicator Cluster Surveys (MICS). The primary data were collected at the household level from women aged 15–49 years. The HEM data repository contains data from nearly 250 DHS and MICS conducted in 94 countries during 1993–2013 (Table 1); almost three-quarters of these countries had surveys available from at least two time points. The data repository covers 34 RMNCH indicators, which are grouped by specified themes. The tables of the repository can be filtered according to indicator, dimension of inequality, country, year and data source.

**Table 1.** Countries included in Health Equity Monitor, by World Health Organization Region

Country	Number of surveys	Survey source(s) and year(s)
**African Region**		
Benin	4	DHS 2011–12, DHS 2006, DHS 2001, DHS 1996
Burkina Faso	4	DHS 2010, MICS 2006, DHS 2003, DHS 1998–99
Burundi	2	DHS 2010, MICS 2005
Cameroon	4	DHS 2011, MICS 2006, DHS 2004, DHS 1998
Central African Republic	3	MICS 2010, MICS 2006, DHS 1994–95
Chad	2	DHS 2004, DHS 1996–97
Comoros	2	DHS 2012, DHS 1996
Congo	2	DHS 2011–12, DHS 2005
Côte d'Ivoire	4	DHS 2011–12, MICS 2006, DHS 1998–99, DHS 1994
Democratic Republic of the Congo	3	DHS 2013–14, MICS 2010, DHS 2007
Ethiopia	3	DHS 2011, DHS 2005, DHS 2000
Gabon	2	DHS 2012, DHS 2000
Gambia	1	MICS 2005–06
Ghana	6	MICS 2011, DHS 2008, MICS 2006, DHS 2003, DHS 1998, DHS 1993
Guinea	3	DHS 2012, DHS 2005, DHS 1999
Guinea-Bissau	1	MICS 2006
Kenya	4	DHS 2008–09, DHS 2003, DHS 1998, DHS 1993
Lesotho	2	DHS 2009, DHS 2004
Liberia	2	DHS 2013, DHS 2007
Madagascar	3	DHS 2008–09, DHS 2003–04, DHS 1997
Malawi	4	DHS 2010, MICS 2006, DHS 2004, DHS 2000
Mali	4	DHS 2012–13, DHS 2006, DHS 2001, DHS 1995–96
Mauritania	1	MICS 2007
Mozambique	4	DHS 2011, MICS 2008, DHS 2003, DHS 1997
Namibia	2	DHS 2006–07, DHS 2000
Niger	3	DHS 2012, DHS 2006, DHS 1998
Nigeria	6	DHS 2013, MICS 2011, DHS 2008, MICS 2007, DHS 2003, DHS 1999
Rwanda	3	DHS 2010, DHS 2005, DHS 2000
Sao Tome and Principe	1	DHS 2008–09
Senegal	4	DHS 2012–13, DHS 2010–11, DHS 2005, DHS 1997
Sierra Leone	4	DHS 2013, MICS 2010, DHS 2008, MICS 2005
South Africa	1	DHS 1998
Swaziland	2	MICS 2010, DHS 2006–07
Togo	3	MICS 2010, MICS 2006, DHS 1998
Uganda	4	DHS 2011, DHS 2006, DHS 2000–01, DHS 1995
United Republic of Tanzania	4	DHS 2010, DHS 2004–05, DHS 1999, DHS 1996
Zambia	3	DHS 2007, DHS 2001–02, DHS 1996
Zimbabwe	5	DHS 2010–11, MICS 2009, DHS 2005–06, DHS 1999, DHS 1994
**Region of the Americas**		
Belize	2	MICS 2011, MICS 2006
Bolivia (Plurinational State of)	4	DHS 2008, DHS 2003, DHS 1998, DHS 1994
Brazil	1	DHS 1996
Colombia	4	DHS 2010, DHS 2005, DHS 2000, DHS 1995
Costa Rica	1	MICS 2011
Cuba	2	MICS 2010–11, MICS 2006
Dominican Republic	4	DHS 2007, DHS 2002, DHS 1999, DHS 1996
Guatemala	2	DHS 1998–99, DHS 1995
Guyana	2	DHS 2009, MICS 2006
Haiti	4	DHS 2012, DHS 2005–06, DHS 2000, DHS 1994–95
Honduras	2	DHS 2011–12, DHS 2005–06
Jamaica	1	MICS 2005
Nicaragua	2	DHS 2001, DHS 1997
Peru	7	DHS 2012, DHS 2011, DHS 2010, DHS 2009, DHS 2006, DHS 2000, DHS 1996
Suriname	2	MICS 2010, MICS 2006
Trinidad and Tobago	1	MICS 2006
**Eastern Mediterranean Region**		
Afghanistan	1	MICS 2010–11
Djibouti	1	MICS 2006
Egypt	4	DHS 2008, DHS 2005, DHS 2000, DHS 1995
Iraq	2	MICS 2011, MICS 2006
Jordan	4	DHS 2012, DHS 2007, DHS 2002, DHS 1997
Morocco	1	DHS 2003–04
Pakistan	2	DHS 2012–13, DHS 2006–07
Somalia	1	MICS 2006
Syrian Arab Republic	1	MICS 2006
Yemen	1	MICS 2006
**European Region**		
Albania	2	DHS 2008–09, MICS 2005
Armenia	3	DHS 2010, DHS 2005, DHS 2000
Azerbaijan	1	DHS 2006
Belarus	2	MICS 2012, MICS 2005
Bosnia and Herzegovina	2	MICS 2011–12, MICS 2006
Georgia	1	MICS 2005
Kazakhstan	4	MICS 2010–11, MICS 2006, DHS 1999, DHS 1995
Kyrgyzstan	3	DHS 2012, MICS 2005–06, DHS 1997
Montenegro	1	MICS 2005–06
Republic of Moldova	1	DHS 2005
Serbia	2	MICS 2010, MICS 2005
Tajikistan	2	DHS 2012, MICS 2005
The former Yugoslav Republic of Macedonia	2	MICS 2011, MICS 2005–06
Turkey	3	DHS 2003, DHS 1998, DHS 1993
Ukraine	2	DHS 2007, MICS 2005
Uzbekistan	2	MICS 2006, DHS 1996
**South-East Asia Region**		
Bangladesh	7	DHS 2011, DHS 2007, MICS 2006, DHS 2004, DHS 1999–2000, DHS 1996–97, DHS 1993–94
Bhutan	1	MICS 2010
India	2	DHS 2005–06, DHS 1998–99
Indonesia	5	DHS 2012, DHS 2007, DHS 2002–03, DHS 1997, DHS 1994
Maldives	1	DHS 2009
Nepal	5	MICS 2010, DHS 2011, DHS 2006, DHS 2001, DHS 1996
Thailand	1	MICS 2005–06
Timor-Leste	1	DHS 2009–10
**Western Pacific Region**		
Cambodia	3	DHS 2010, DHS 2005, DHS 2000
Lao People's Democratic Republic	2	MICS 2011–12, MICS 2006
Mongolia	2	MICS 2010, MICS 2005
Philippines	5	DHS 2013, DHS 2008, DHS 2003, DHS 1998, DHS 1993
Vanuatu	1	MICS 2007
Viet Nam	4	MICS 2010–11, MICS 2006, DHS 2002, DHS 1997

The HEM theme page supports the interpretation and reporting of the data from the repository. It contains a range of resources such as:

- interactive visuals (available for selected RMNCH sub-themes) that allow users to customize graphical displays of the data;
- feature stories that provide a comprehensive assessment of a selected health indicator and dimension of inequality;
- equity country profiles that highlight all available data for a given country in a customizable display;
- and links to publications, reports and tools that relate to the content of the repository.

Technical information about the data, including detailed indicator definitions, are accessible from the data repository or through the theme page. Regular updates to the HEM data repository and theme page are done approximately once per year to expand the database with newly available data and update the associated texts and visuals.

## Data collected

The source data for HEM consist of micro-data from publicly available DHS and MICS. The USAID-supported DHS and UNICEF-supported MICS are the two largest global household survey programmes that collect data about RMNCH. The data collection strategy for DHS and MICS has been detailed previously.^1^^,^^2^ Briefly, DHS and MICS are repeated, cross-sectional, national surveys that collect data at the household level, including standardized, face-to-face interviews with women. DHS and MICS cover a range of health topics and socio-demographic characteristics. The health indicators and dimensions of inequality currently featured in the HEM are shown in Tables 2 and 3. The source data are freely available through [www.measuredhs.com] (DHS) and [www.childinfo.org] (MICS). Although there are minor differences between the survey tools used by DHS and MICS (as elaborated upon in the ‘Strengths and weaknesses’ section below), the surveys are assumed to be sufficiently similar to permit direct comparisons between surveys, across countries and over time. (DHS and MICS work together to ensure that their tools are harmonized and comparable for the purposes of creating global databases that cover a large number of low- and middle-income countries).^1^

**Table 2.** Reproductive, maternal, newborn and child health indicators included in Health Equity Monitor

Indicator category	Indicator
**Health intervention indicators**	
Reproductive health interventions	Contraceptive prevalence: modern and traditional methods (%)
	Contraceptive prevalence: modern methods (%)
	Demand for family planning satisfied (%)
Maternal health interventions	Antenatal care coverage: at least four visits (%)^a^
	Antenatal care coverage: at least one visit (%)^a^
	Births attended by skilled health personnel (%)^a^
	Births by caesarean section (%)^a^
	Pregnant women sleeping under insecticide-treated nets (%)
Newborn and child health interventions	BCG immunization coverage among 1-year-olds (%)
	Children aged 6–59 months who received vitamin A supplementation (%)
	Children aged <5 years sleeping under insecticide-treated nets (%)
	Children aged <5 years with diarrhoea receiving oral rehydration salts (%)
	Children aged <5 years with diarrhoea receiving oral rehydration therapy and continued feeding (%)
	Children aged <5 years with pneumonia symptoms taken to a health facility (%)
	DTP3 immunization coverage among 1-year-olds (%)
	Early initiation of breastfeeding (%)^a^
	Full immunization coverage among 1-year-olds (%)
	Measles immunization coverage among 1-year-olds (%)
	Polio immunization coverage among 1-year-olds (%)
RMNCH interventions, combined	Composite coverage index (%)
**Health outcome indicators**	
Obesity in non-pregnant women	Prevalence of obesity in non-pregnant women aged 15–49 years, BMI >= 30 (%)
Fertility	Adolescent fertility rate (per 1000 women aged 15–19 years)
	Total fertility rate (per woman)
Child malnutrition	Stunting prevalence in children aged <3 years (%)
	Stunting prevalence in children aged <5 years (%)
	Underweight prevalence in children aged <3 years (%)
	Underweight prevalence in children aged <5 years (%)
	Wasting prevalence in children aged <3 years (%)
	Wasting prevalence in children aged <5 years (%)
Child mortality	Infant mortality rate (deaths per 1000 live births)
	Neonatal mortality rate (deaths per 1000 live births)
	Under-five mortality rate (deaths per 1000 live births)

^a^DHS data are based on the 3 years or 5 years preceding survey and MICS data are based on the 2 years preceding survey. A complete compendium of details about indicator definitions is available from [http://www.who.int/gho/health_equity/outcomes/health_equity_compendium.pdf?ua=1].

**Table 3.** Dimensions of inequality represented in Health Equity Monitor

Dimension of inequality	Notes about measurement and application
Education	Measured as three subgroups (no education, primary school and secondary school or higher). In the case of newborn and child health indicators, the mother’s level of education is applied
Economic status	Measured as household wealth quintiles, derived from asset indices. Country-specific asset indices were based on owning selected assets and having access to certain services, and were constructed using principal component analysis
Place of residence	Measured as rural or urban, according to national criteria
Subnational region	Measured according to national criteria
Child’s sex	Child’s sex is applied to indicators of newborn and child health

For the purposes of HEM, micro-data from DHS and MICS were re-analysed by ICEH [www.equidade.org] through a process that has been previously applied to initiatives such as the Countdown to 2015 country profiles.^3^ An analysis platform was developed at ICEH that allows for batch analysis of DHS and MICS surveys using a single standard code. This assures that estimates are all based on the same standard definitions, as agreed upon by major international agencies such as WHO. The code can incorporate adaptations allowed to countries, such as local definitions of which professional categories are considered skilled to deliver a baby. After the estimation process, each indicator is thoroughly checked for correctness. Point estimates along with 95% confidence intervals are prepared and formatted to upload in the HEM data repository.

RMNCH was chosen as the current thematic focus for HEM due to its relevance in the area of global health and development, and the relatively high availability of these data across low- and middle-income countries. The indicators were selected for inclusion based on data availability, relevance to the work of other initiatives (such as Countdown to 2015, and the Commission on Information and Accountability for Women’s and Children’s Health) and alignment with the Millennium Development Goals. Indicators represent health interventions and outcomes across the continuum of RMNCH (Table 2). Standardized indicator definitions were applied according to official WHO definitions, with a few exceptions.^4^ Indicators specifying the use of ‘skilled health personnel’ differ from the WHO definition, which employs a uniform definition of skilled health personnel across all countries that includes only doctors, nurses and midwives; HEM definitions are based on all relevant health professionals determined for each country. For child immunization indicators, the reference age group used in the denominator (12–23 months) was adjusted to align with alternative immunization schedules adopted in certain countries (18–29 months or 15–26 months).

The dimensions of inequality represented in HEM are the basis of data disaggregation at the country level, as outlined in Table 3.

## Data resource use

Designed to appeal to both technical and non-technical users, this data resource has four primary uses. First, the data tables contained in the HEM data repository can be downloaded for further analyses of inequality at global or national levels. With these tables of disaggregated data, users can monitor health inequalities, including performing benchmark comparisons between countries of interest and making comparisons based on dimensions of inequality, health indicators and/or survey years. A recently developed WHO resource, the Health Equity Assessment Toolkit (HEAT), promotes and streamlines the expanded use of the HEM database.^5^ HEAT facilitates the assessment of within-country health inequalities through the calculation of inequality summary measures and the production of customized tables and graphs. It is comprised of two components: *Explore inequality*, which enables users to assess the state of inequality within a country of interest; and *Compare inequality*, which enables users to compare the situation in one country of interest with the situation in other countries. Results can be exported from HEAT and used for priority setting and decision making in countries.

A second use of HEM is as a source of reference data for the preparation of reports and publications about health inequalities. The 2015 report *State of Inequality: Reproductive, Maternal, Newborn and Child Health*, for example, used data from HEM to showcase inequalities in 23 RMNCH indicators across 86 low- and middle-income countries.^6^ The report contextualizes the data about the current and past state of inequality by presenting a series of feature stories which were also represented through interactive visuals (storyboards) and video clips. This report and its accompanying electronic components are available on the HEM theme page. In addition, other publications have drawn upon data from HEM to advocate for and illustrate fundamental concepts about-the importance of health inequality monitoring.^7–9^ Country reports about the state of inequality in RMNCH have been prepared using HEM data.^10^^,^^11^

Third, HEM data are used for ongoing monitoring and evaluation of global programmes. One example is the Global Vaccine Action Plan, a framework endorsed by the Member States of the WHO to promote equitable access to existing vaccines for people in all communities. Drawing from HEM data, GVAP monitors inequalities on the basis of indicators that address within-country inequality, including a measure that compares DTP3 coverage (three doses of the combined diphtheria, tetanus toxoid and pertussis vaccine) in the country’s poorest wealth quintile with the level in the richest wealth quintile.^12^

Fourth, through the contents of the theme page, HEM serves as a guide for the application of disaggregated data for the purposes of health inequality monitoring. The HEM theme page contains concise and accurate messaging relevant to HEM data, highlighting entry points for further exploration of the data tables. For example, interactive visuals illustrate patterns of inequality across countries for selected indicators, and ‘Read more’ features demonstrate components of high-quality reporting. Furthermore, the HEM theme page contains resources such as the *Handbook on Health Inequality Monitoring: With a Special Focus on Low- And Middle-Income Countries,* which outlines the steps for monitoring health inequalities and provides practical advice.^9^ A complementary *Health Inequality Monitoring eLearning Module* was created to reinforce the content of the *Handbook* by promoting theoretical understanding and practical application of health inequality monitoring through a learner-oriented tool.^13^

## Strengths and weaknesses

This data resource is one of the largest repositories of disaggregated data from low- and middle-income countries on the subject of RMNCH. Through the data repository and theme page, HEM provides open access to data that are suitable for health inequality monitoring, and also comprehensive information about the data, including detailed indicator definitions. To our knowledge, HEM is the only global health database that reports 95% confidence intervals for disaggregated data. Additionally, HEM contains a wealth of resources to promote and demonstrate high-quality data analysis and reporting. In particular, communicating stories from the data through interactive visualization is a unique feature of HEM that supports data interpretation and exploration by a range of potential audiences.

The use of primary data from DHS and MICS has several advantages. Notably, DHS and MICS are widely administered internationally, enabling the inclusion of 94 countries in HEM, and DHS and MICS are conducted on a repeating basis, permitting frequent updates to HEM.

Limitations of the HEM include the scope of data represented, in terms of the health topic and country representation. HEM currently contains data pertaining to RMNCH, though the availability of disaggregated data for global health inequality monitoring across other health topics is recognized as an emerging priority.^14^ At present, for the sake of comparability between countries and over time, HEM only contains disaggregated data from international DHS and MICS. As a result, HEM lacks data from countries that do not participate in DHS or MICS, including most high-income countries. In addition, several large upper-middle income countries either have never participated in DHS or MICS (such as China and Mexico), or do not have a recent survey (i.e. the last survey was carried out a decade or more ago) (such as Brazil and South Africa).

For certain maternal health indicators, the data collection protocols for DHS and MICS specify different time periods (see Table 2). These indicators reflect experiences in the 3 or 5 years before the survey for DHS, and the 2 years before the survey for MICS. For transparency, the HEM data repository displays these data as separate entries.

In some cases, estimates based on low sample sizes pose a limitation. Disaggregation of the sample population (e.g. into wealth quintiles) necessarily decreases the sample size of the point estimate (e.g. for each wealth quintile). To address this issue, the HEM data repository flags cases where the estimate is based on low sample sizes (e.g. 25–49 cases for RMNCH intervention indicators) and where the estimate is not reported due to prohibitively low sample size (e.g. fewer than 25 cases for RMNCH intervention indicators).

For data that are disaggregated by subnational region, HEM retains the original subnational region names from DHS and MICS; in some cases, there may be differences in the spelling and/or number of subnational regions between survey years.

Profile in a nutshell

- The Health Equity Monitor (HEM) was set up to enable global or national health inequality monitoring, especially in low- and middle-income countries. Featuring data about reproductive, maternal, newborn and child health (RMNCH) indicators, it is one of the largest repositories of disaggregated data that are comparable across countries and over time.
- Launched in 2013, HEM covers nearly 250 household health surveys conducted in 94 countries from all world regions, spanning 1993–2013. Regular updates to the HEM are done approximately once per year.
- HEM is a repository of re-analysed micro-data from Demographic and Health Surveys (DHS) and Multiple Indicator Cluster Surveys (MICS). DHS and MICS are large-scale, nationally representative household surveys that collect data from women aged 15–49 years through standardized, face-to-face interviews.
- HEM includes RMNCH indicators, disaggregated by education, economic status, place of residence (rural/urban), subnational region and child’s sex (where applicable).
- HEM is a collaboration between the World Health Organization Department of Information, Evidence and Research (Geneva, Switzerland); the World Health Organization Gender, Equity and Human Rights Team (Geneva, Switzerland); and the International Center for Equity in Health based in the Federal University of Pelotas (Pelotas, Brazil). All data in HEM are openly available through the Global Health Observatory of the World Health Organization [apps.who.int/gho/data/node.main.HE-1540?lang=en]. The HEM theme page highlights key messages from the data repository, and includes interactive visuals and feature stories [www.who.int/gho/health_equity/en/].

## Data resource access

All data tables in HEM are openly available through the Global Health Observatory of the WHO [apps.who.int/gho/data/node.main.HE-1540?lang=en]*.* Data tables can be filtered based on any combination of the following variables: indicator, dimension of inequality, country, year and data source. Quick download options are available for data tables that have been filtered, generating a multipurpose table in Excel or CSV format. Full versions of the data tables can be downloaded from the data repository in CSV, Excel, HTML, JSON and XML formats, with additional formatting options such as simplified or complete structure, and tables or lists. All components of the HEM theme page are accessible through [www.who.int/gho/health_equity/en/].

A complete compendium of definitions of HEM indicators is available as a PDF [http://www.who.int/gho/health_equity/outcomes/health_equity_compendium.pdf?ua=1]. Alternatively, indicator definitions can be accessed through the WHO Indicator and Measurement Registry of the Global Health Observatory [http://www.who.int/gho/indicator_registry/en/]; definitions can be exported from the Registry in SDMX-HD format.

**Conflict of interest:** None declared.
